# Insecticidal Activity of a Vip3Ab1 Chimera Is Conferred by Improved Protein Stability in the Midgut of *Spodoptera eridania*

**DOI:** 10.3390/toxins11050276

**Published:** 2019-05-16

**Authors:** Andrew J. Bowling, Megan S. Sopko, Sek Yee Tan, Cory M. Larsen, Heather E. Pence, Marc D. Zack

**Affiliations:** Corteva Agriscience, Indianapolis, IN 46268, USA; msopko@indianabiosciences.org (M.S.S.); sekyee.tan@bayer.com (S.Y.T.); cory.larsen@corteva.com (C.M.L.); heather.pence@corteva.com (H.E.P.); marc.zack@genective.com (M.D.Z.)

**Keywords:** Vip3A, spodoptera, Bt, *Bacillus thuringiensis*, insect histopathology, immunolocalization, lepidoptera

## Abstract

Vip3A proteins are important for the control of spodopteran pests in crops, including *Spodoptera frugiperda* (fall armyworm). Native Vip3Ab1 controls *S. frugiperda*, but it is ineffective against *S. eridania* (southern armyworm), a major pest of soybean in South America. Recently, a Vip3Ab1 chimera with a modified C-terminus was described, Vip3Ab1-740, which has increased potency against *S. eridania* while maintaining activity against *S. frugiperda*. As *S. frugiperda* and *S. eridania* are differentially susceptible to Vip3Ab1, experiments were conducted to identify and understand the mechanism by which this expanded potency is conferred. The role of protein stability, processing, and in vivo effects of Vip3Ab1 and Vip3Ab1-740 in both of these species was investigated. Biochemical characterization of the midgut fluids of these two species indicated no obvious differences in the composition and activity of digestive enzymes, which protease inhibitor studies indicated were likely serine proteases. Histological examination demonstrated that both proteins cause midgut disruption in *S. frugiperda*, while only Vip3Ab1-740 affects *S. eridania*. Immunolocalization indicated that both proteins were present in the midgut of *S. frugiperda*, but only Vip3Ab1-740 was detected in the midgut of *S. eridania*. We conclude that the gain of toxicity of Vip3Ab1-740 to *S. eridania* is due to an increase in protein stability in the midgut, which was conferred by C-terminal modification.

## 1. Introduction

Vegetative insecticidal proteins (Vip) are produced by *Bacillus thuringiensis* (Bt) as soluble proteins during the vegetative phase of their life cycle, and are valued for their broad-spectrum activity against lepidopteran pests [1,2]. Vip3A proteins are generally accepted to be pore-forming proteins bearing some similarity to the more described Cry family of insecticidal proteins. Vip3A proteins associate as tetramers requiring proteolytic processing prior to insecticidal pore-forming activity [3,4,5]. The precise molecular mechanism by which pore formation occurs has not been fully elucidated, but is thought to involve specific binding to cellular receptors on the midgut epithelium [1]. Importantly, in vitro studies demonstrate that Vip3A proteins do not compete with Cry proteins for binding sites on brush border membrane vesicles (BBMV) and in vivo studies have shown Vip3A proteins maintain potent insecticidal activity against Cry-resistant insects [6,7,8,9,10,11]. Thus, Vip3A is especially important for control of *S. frugiperda* (fall armyworm, FAW), which has documented resistance to first generation transgenic crops containing the Bt crystal proteins, Cry1Fa and Cry1Ab [12,13,14,15,16,17]. Interestingly, Vip3A has differing levels of toxicity to various spodopteran insects; for example, native Vip3Ab1 has potent lethal activity on *S. frugiperda*, but has very little effect on *S. eridania* (southern armyworm, SAW).

It is generally thought that Vip3A proteins bind specific membrane receptors that are different than those of Cry proteins. However, despite the proposal of multiple Vip3A receptor candidates, a definitive receptor has not yet been demonstrated. In fact, receptor binding does not appear to be the sole discriminatory step in Vip3A-mediated insecticidal activity, as membrane preparations from susceptible, non-susceptible, and resistant insects have demonstrated similar specific binding [10,18]. Furthermore, midgut fluids from non-susceptible insects contain an enzymatic profile similar to susceptible insects, with equivalent capability to process Vip3A precursors to their putative active form [10]. Therefore, an in-depth understanding of the mechanisms that govern Vip3A insecticidal susceptibility or resistance is important for maintaining the value of Vip3A as an effective insecticidal component in lepidopteran pest management strategies. 

There are approximately 100 members of the Vip3A gene family, which all share at least 78% identity at the amino acid level. However, similarity is not balanced over the length of the protein, as the diversity increases towards the C-terminus [1,19]. This observation has led several groups to hypothesize that the C-terminus determines Vip3A specificity and, in fact, our group has shown that replacement of the final 580 amino acids of Vip3Bc1 with the corresponding region of Vip3Ab1 results in activity towards insects susceptible to Vip3Ab1 [5]. Recently, we utilized this information to make more modest modifications to the native C-terminal 177 amino acids of Vip3Ab1 to produce a chimeric protein, Vip3Ab1-740 [20]. Interestingly, this new protein has lethal activity on both *S. frugiperda* and *S. eridania*, demonstrating that the C-terminal portion of the protein contributes to the insecticidal spectrum of Vip3Ab1. However, the specific mechanism for this increased spodopteran spectrum is not currently known. Differential membrane binding utilizing brush border membrane vesicles (BBMV) has not been demonstrated between any members of the Vip3A family, and BBMV derived from Vip3A-resistant insects show Vip3A binding that is similar to BBMV from susceptible insects [8,18]. Due to the existing data indicating that Vip3A specificity may not be entirely dependent on membrane binding, we investigated alternative hypotheses that Vip3Ab1-740 activity towards *S. eridania* was conferred by other biochemical attributes making this Vip3A chimera more stable in the *S. eridania* midgut. We investigated the rate of proteolytic processing by midgut fluids from *S. eridania* and *S. frugiperda*. We also evaluated the relative stability of Vip3A tetramers by analytical size exclusion chromatography, which indicated that Vip3Ab1-740 had increased in vitro stability relative to Vip3Ab1. Finally, we designed experiments to evaluate Vip3A protein stability in vivo, including co-feeding with proteinase inhibitor and then direct in situ visualization utilizing histology and immunolocalization. Results from these experiments are the first to directly demonstrate that differential Vip3A spectrum can be conferred via increased protein stability in the target insect midgut.

## 2. Results

### 2.1. Protein Characterization by Midgut Fluid Digestion and Analytical Size Exclusion Chromatography

**Figure 1:**
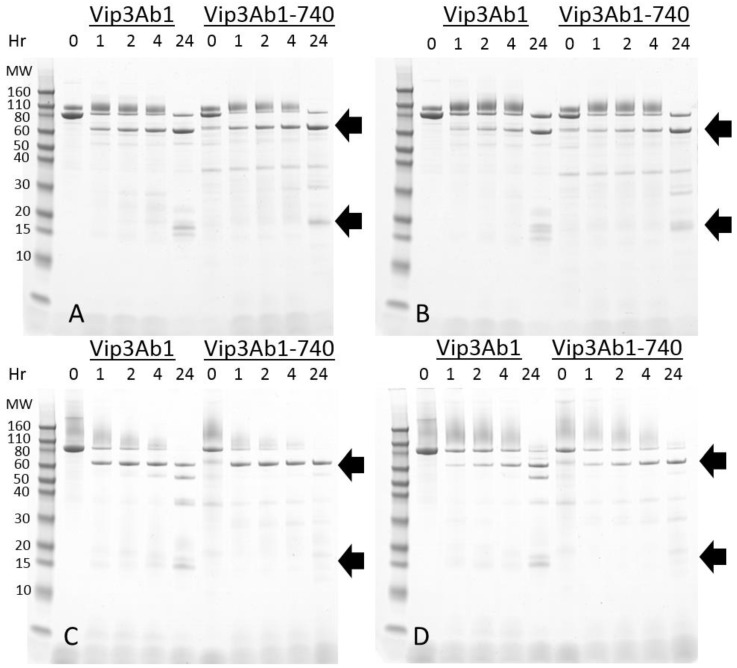
SDS-PAGE analysis of time course digestion of Vip3Ab1 and Vip3Ab1-740. (**A**) *S. frugiperda* gut fluid digestion, pH 8.0. (**B**) *S. eridania* gut fluid digestion, pH 8.0. (**C**) *S. frugiperda* gut fluid digestion, pH 10.0. (**D**) *S. eridania* gut fluid digestion, pH 10.0. Reactions were incubated for various time intervals at 30 °C and terminated with protease inhibitors prior to SDS-PAGE analysis.

To compare the rate of proteolytic processing of Vip3Ab1 and Vip3Ab1-740 by *S. frugiperda* and *S. eridania* midgut fluids, a time course digestion was performed and products were analyzed by SDS-PAGE (Figure 1). Reactions were performed at both pH 8.0 and pH 10.0. The amount of *S. frugiperda* and *S. eridania* midgut fluids added to each reaction was normalized by total proteolytic activity, as previously described [5]. Both Vip3Ab1 and Vip3Ab1-740 were processed to ~65 kDa and ~20 kDa products (Figure 1, arrows) at similar rates, by either *S. frugiperda* or *S. eridania* midgut fluids. At pH 8.0, a portion of full-length protein remained visible for both proteins after 24 h digestion by either *S. frugiperda* or *S. eridania* midgut fluids. However, at pH 10.0, the reactions proceeded more rapidly and very little full-length protein remained after 24 h digestion. At 24 h, pH 10.0, the ~65 kDa product of Vip3Ab1 appeared to be partially degraded into additional smaller sized products, a phenomenon which was not observed in Vip3Ab1-740. The ~20 kDa products from both proteins are not well resolved after overnight incubation in either pH. However, in some cases the ~20 kDa overnight products appear to contain multiple bands, which may indicate degradation at either the N- or C-terminus. The presence of multiple bands at ~20 kDa appears more pronounced in Vip3Ab1 than in Vip3Ab1-740. 

**Figure 2:**
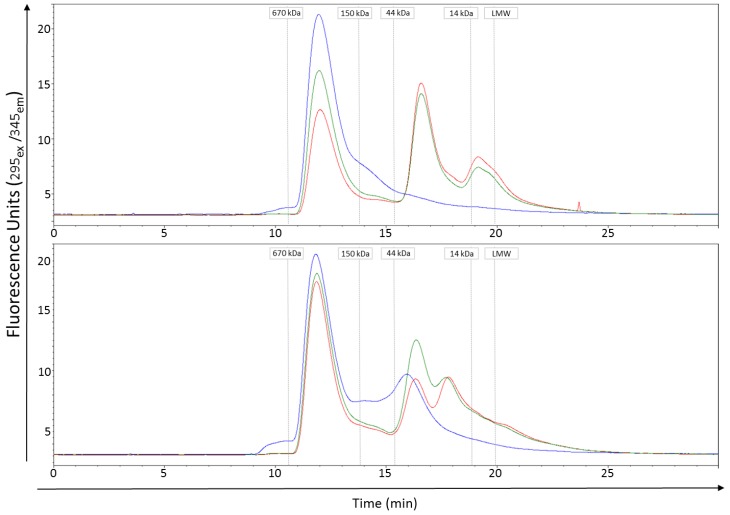
Size exclusion chromatography of Vip3Ab1 (top panel) and Vip3Ab1-740 (bottom panel). Both proteins had a main peak with a retention time of ~11.9 min before (blue) and after *S. frugiperda* (red) and *S. eridania* (green) midgut fluid digestion that was consistent with a tetrameric state based on molecular weight standards. Digestions were performed for overnight at pH 10.0. A smaller peak at ~15.9 min in the full length Vip3Ab1-740 may represent truncated material. Additional peaks in the midgut fluid digestions at ~16.6 and ~19.1 min (Vip3Ab1) and ~16.3 and ~17.8 min (Vip3Ab1-740) indicate smaller sized degradation products. Retention times of molecular weight standards (gray lines) are included for reference.

As SDS-PAGE suggested differential stability of Vip3Ab1 and Vip3Ab1-740, we employed analytical size exclusion chromatography to evaluate tetramer stability in native conditions, as Vip3Ab1 forms tetramers both before and after digestion with lepidopteran midgut fluids [5]. Chromatograms from overnight incubations with midgut fluids from either *S. frugiperda* or *S. eridania* (or no midgut fluids) are shown in Figure 2. Both proteins had a main peak with a retention time of ~11.9 min with and without midgut fluid, which is consistent with the presence of protein tetramers. As SDS-PAGE indicates, both full length proteins are almost entirely degraded after overnight digestion (Figure 1). Thus, the peaks observed in the midgut fluid-digested proteins in Figure 2 correspond to processed ~65 and ~20 kDa products that remain associated after midgut fluid digestion. The main tetrameric peak for Vip3Ab1 decreased in size as much as 50% in the midgut fluid digested samples compared to the full length, non-digested sample. Additional peaks appeared at later retention times (~16.5 and ~19.1 min), corresponding to smaller sized degradation products. This observation is consistent with the partial breakdown of the ~65 kDa product into smaller sized products at pH 10.0 after 24 h of digestion observed by SDS-PAGE (Figure 1). Full-length, non-digested Vip3Ab1-740 had an additional smaller peak at ~15.9 min (Figure 2), indicating that a small amount of truncated protein is present. Vip3Ab1-740 also had only a small reduction in size of the main tetrameric peak in the midgut fluid digested samples compared to the untreated full-length protein. However, some smaller sized degradation products were observed at ~16.3 and ~17.8 min.

### 2.2. Characterization of Proteases Present in S. frugiperda and S. eridania Midgut Fluids

**Figure 3:**
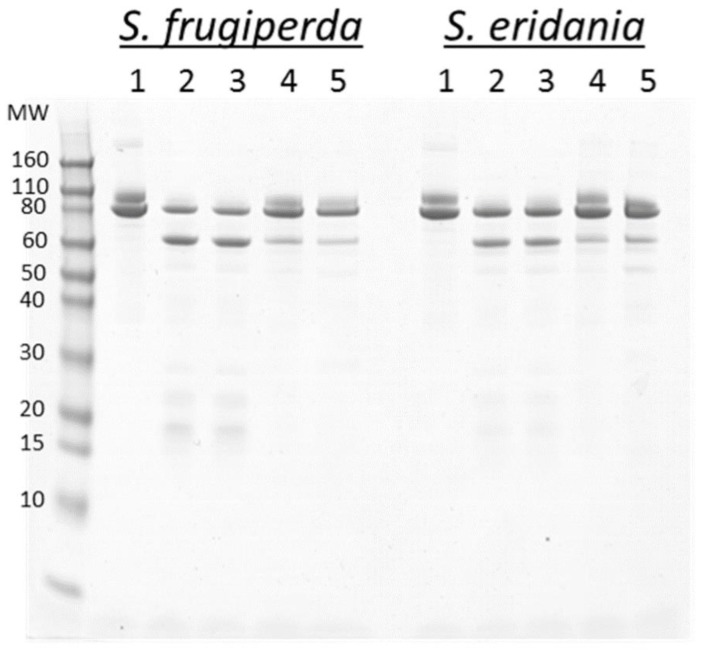
SDS-PAGE analysis of overnight digestion of Vip3Ab1 with *S. frugiperda* and *S. eridania* gut fluids at pH 8.0 with the addition of bestatin, chymostatin and benzamidine in separate reactions. Control reactions with no enzyme and no inhibitors also were included. Lanes: (1) No enzyme, (2) no inhibitor, (3) bestatin, (4) chymostatin, (5) benzamidine. All reactions were terminated with protease inhibitors prior to SDS-PAGE analysis.

In order to identify the class of proteases present in *S. frugiperda* and *S. eridania* midgut fluids, Vip3Ab1 was digested at pH 8.0 overnight with each midgut fluid in the presence of different protease inhibitors and analyzed by SDS-PAGE (Figure 3). The results show no obvious differences in digestion products between the two species in the presence of these inhibitors. The addition of bestatin, an aminopeptidase inhibitor, yielded results similar to the no inhibitor control. The addition of chymostatin, a specific inhibitor of α-, β-, γ- and δ-chymotrypsin, inhibited the processing of Vip3Ab1. The addition of benzamidine, a general serine protease inhibitor, also inhibited the processing of Vip3Ab1. *S. frugiperda* and *S. eridania* midgut fluids were also analyzed by casein zymogram to compare the banding patterns of proteases, and the two midgut fluids showed a similar pattern of negatively stained bands (Figure S1).

### 2.3. Effect of Protease Inhibitors on In-Vivo Diet Bioassay

**Figure 4:**
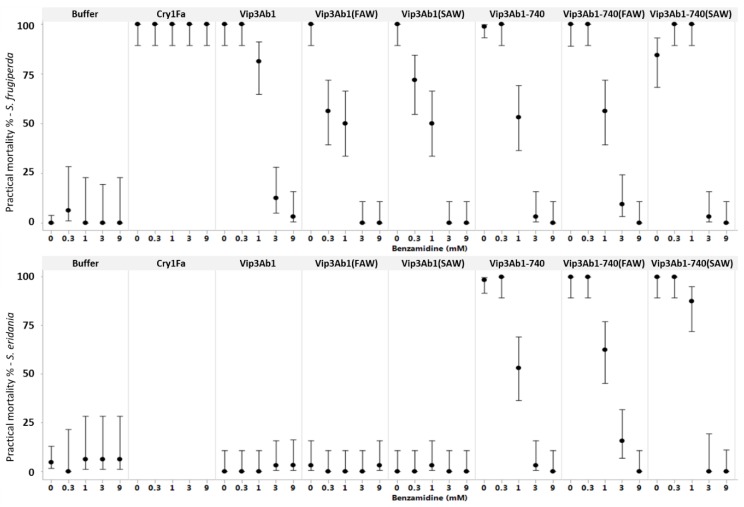
Evaluation of benzamidine effects on insecticidal protein activity against *S. frugiperda* (top) and *S. eridania* (bottom). All treatments were assayed by diet overlay at 3000 ng/cm^2^. Full length Vip3Ab1 and Vip3Ab1-740 and identical samples pre-processed in vitro by *S. frugiperda* (FAW) or *S. eridania* (SAW) midgut enzymes were assayed in the presence of 0, 0.3, 1, 3 or 9 mM benzamidine. Cry1Fa was also included for comparison utilizing *S. frugiperda*. Results are displayed as percent practical mortality, which includes dead and moribund insects, with upper and lower 95% confidence intervals.

Since benzamidine was shown to slow the proteolytic processing of both Vip3Ab1 and Vip3Ab1-740 when incubated with *S. frugiperda* or *S. eridania* midgut fluids (Figure 3), we further investigated this effect in vivo. First, a concentration series of benzamidine demonstrated that this inhibitor is not toxic to either species (Figure 4, left). Next, benzamidine was added at various concentrations, in combination with either full length or gut fluid-processed Vip3A proteins at 3000 ng/cm^2^, to test the effect of serine protease inhibition in *S. frugiperda* and *S. eridania*. A shift in LC_50_ values, indicating a decrease in potency, was seen for both gut fluid-processed proteins in the absence of inhibitor (Table 1). Neither full length nor processed Vip3Ab1 was active against *S. eridania*, regardless of benzamidine concentration. Unexpectedly, when benzamidine was co-fed with Vip3Ab1 to *S. frugiperda*, or with Vip3Ab1-740 to both *S. frugiperda* and *S. eridania*, the insecticidal activity of the proteins dramatically decreased in a dose-dependent manner, and activity was completely lost at the highest benzamidine concentration (Figure 4). This inhibition was also observed for gut-fluid processed proteins (Figure 4). For comparison, benzamidine was also tested with Cry1Fa, a three domain Cry protein with known activity against *S. frugiperda,* at 3000 ng/cm^2^, with the same benzamidine concentrations (Figure 4). In this case, all bioassay samples had 100% mortality, regardless of benzamidine concentration.

### 2.4. Histopathological Evaluation of Vip3Ab1 and VipAb1-740 Effects on S. eridania and S. frugiperda

**Figure 5:**
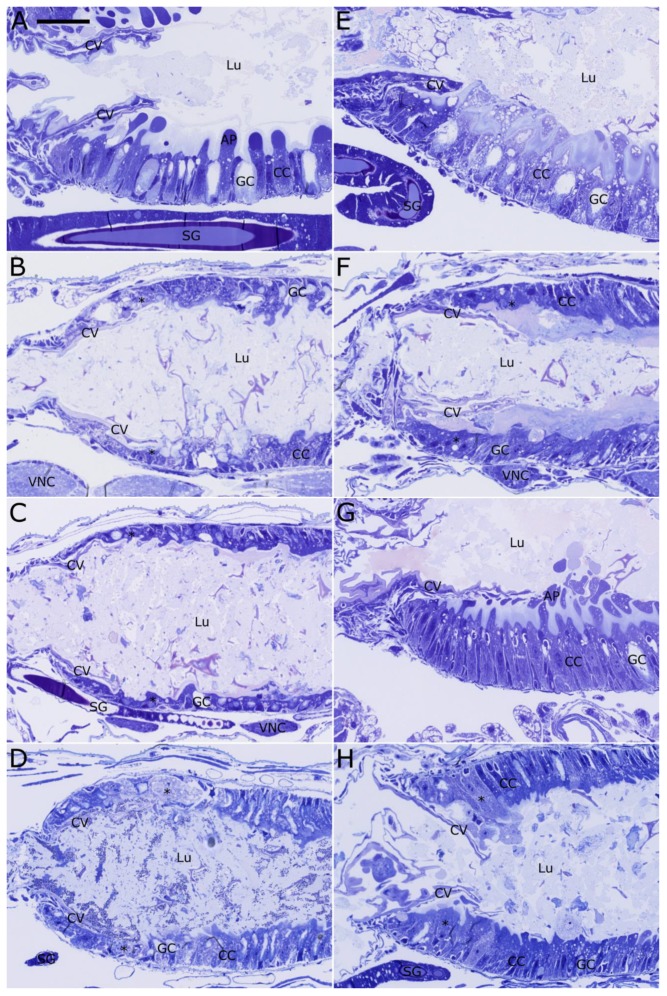
Longisections of the anterior midgut (AMG) region of FAW (**A**–**D**) and SAW (**E**–**H**) either untreated, or after feeding on diet containing one of three toxic proteins for 48 h. (**A**) Untreated FAW, showing the normal appearance of the midgut enterocytes. (**B**) FAW after feeding on Cry1F, showing dramatic damage to the midgut enterocytes, both columnar and goblet cell types. (**C**) FAW after feeding on Vip3Ab1, showing a degree and type of damage that appears very similar to Cry1F. (**D**) FAW after feeding on Vip3Ab1-740, showing a degree and type of damage that appears very similar to Cry1F and Vip3Ab1. (**E**) Untreated SAW, showing the normal appearance of the midgut enterocytes. (**F**) SAW after feeding on Cry1F, showing dramatic damage to the midgut enterocytes, both columnar and goblet cell types. (**G**) SAW after feeding on Vip3Ab1, showing no obvious damage to either columnar or goblet cells. (**H**) SAW after feeding on Vip3Ab1-740, showing obvious damage to the columnar cells. Specifically, the apical protrusions of the columnar cells have been damaged/lost and are no longer visible. The cells in the region directly adjacent to the CV show the most obvious signs of damage, as these cells have begun to lyse and slough off of the basement membrane. (Lu = gut lumen; GC = goblet cells; CC = columnar cells; * = microvillar membrane; AP = apical protrusions; MT = malpighian tubules; arrowheads = stem cells; arrows = circular muscles; scale bar = 25 µm).

A histopathological study was conducted in order to more fully understand the differential impact of these two closely-related proteins on the midgut cells and tissues of *S. frugiperda* and *S. eridania*. The anterior midgut (AMG) of the untreated (UT) *S. frugiperda* shows the normal appearance of the enterocytes in this region (Figure 5A). The columnar cells are relatively long and thin, with smooth, rounded apical protrusions surrounded with a dense layer of microvilli. The goblet cells are somewhat shorter, with open lumens lined with microvilli, basal nuclei, and a very thin rim of cytoplasm. Some peritrophic matrix material is visible between the apical protrusions of the columnar cells and the midgut lumen. After feeding on diet containing Cry1F for 48 h, significant damage to the AMG can be seen (Figure 5B). The anterior-most enterocytes, starting just inside the cardiac valve (CV) and extending back for most of the field of view, appear to have been nearly totally destroyed. However, even with this extensive damage, the midgut lumen is open and contains food material, the circular muscles lining the AMG are relatively loose, and there are very little signs of stem cell activation or other obvious signs of healing responses. *S. frugiperda* larvae that have fed on diet containing Vip3Ab1 show signs of damage that are very similar to Cry1F (Figure 5C). Specifically, the enterocytes lining the AMG have been nearly totally destroyed and most of the apical contents of these cells have been lost. No signs of constriction of the midgut, or any healing responses are evident. The AMG of *S. frugiperda* larvae that have fed on diet containing Vip3Ab1-740 for 48 h shows damage similar to Cry1F and Vip3Ab1 (Figure 5D). Most of the enterocytes in the AMG region have lysed, and the cell contents have been lost to the lumen. The damage to the *S. frugiperda* midgut caused by Vip3Ab1-740 appears slightly less severe than with Cry1F or Vip3Ab1. 

The normal appearance of the AMG of *S. eridania* is similar to *S. frugiperda*, with some relatively small differences (Figure 5E). *S. eridania* larvae have columnar cells and goblet cells which appear very similar to those in *S. frugiperda*. The columnar cells are long and thin, with large apical protrusions lined with dense microvilli. The goblet cells are shorter, and have lumens lined with microvilli. The midgut lumen of the UT *S. eridania* larva is relatively narrow, not because the circular muscles are particularly contracted, but because of the height/size of the enterocytes. After feeding on Cry1F for 48 h, the enterocytes of the AMG are severely damaged (Figure 5F). While some of the basal portions of the enterocytes are still attached to the basement layer, the apical protrusions of the cells of this region have been totally lost. The enterocytes of the AMG of *S. eridania* larvae that have fed on Vip3Ab1 for 48 h appear very similar to the untreated, indicating that these cells have not been damaged by the protein (Figure 5G). There are no signs of a healing response, which has been hypothesized to be a possible method that insects could use to recover from intoxication and survive [4]. The enterocytes in the AMG of *S. eridania* larvae fed on Vip3Ab1-740 for 48 h show some clear signs of damage (Figure 5H). The cells closest to the CV appear to be very close to complete lysis, and the columnar cells in this region have lost nearly all apical protrusions. 

### 2.5. Immunolocalization of Vip3Ab1 and VipAb1-740 in S. eridania and S. frugiperda

**Figure 6:**
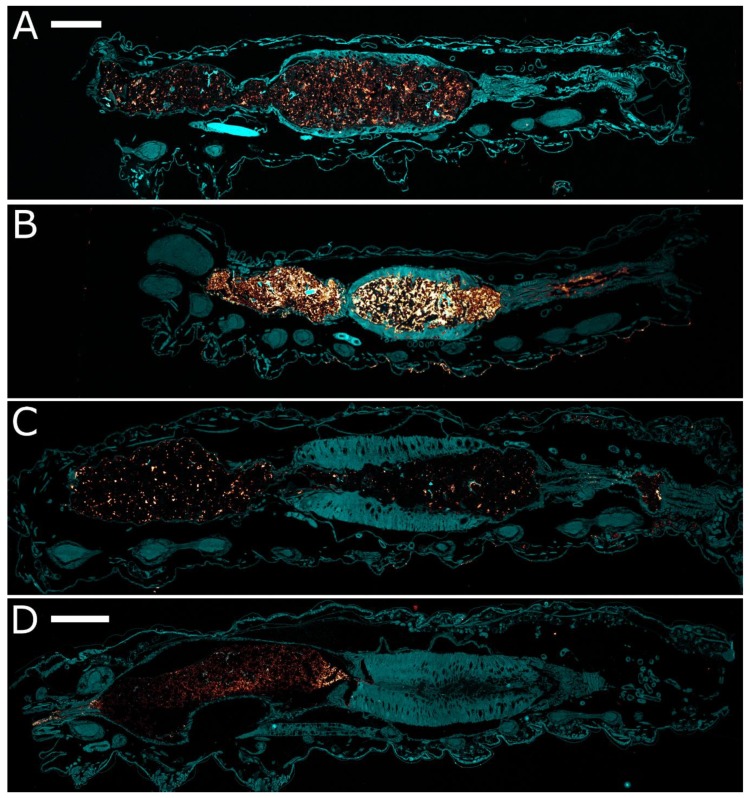
Confocal reflection microscopy of FAW and SAW, comparing Vip3Ab1 and Vip3Ab1-740 localization. (**A**) A FAW larva fed Vip3Ab1-740 for 48 h, showing relatively heavy staining in the lumen of the foregut and midgut. (**B**) A FAW larva fed Vip3Ab1 for 48 h, showing heavy staining in the foregut, midgut and hindgut, and some staining of midgut goblet cell lumens. (**C**) A SAW fed Vip3Ab1-740, showing a relatively small amount of staining in the lumen of the foregut and midgut, but no obvious staining of the midgut cells. (**D**) A SAW fed Vip3Ab1, showing a relatively strong staining of the foregut, but very little staining of the material in the lumen of the midgut. Scale bars—A, B, C = 100 µm, D = 200 µm.

Immunolocalization was conducted to determine if either or both of the Vip3Ab1 and Vip3Ab1-740 proteins were able to cross the peritrophic matrix, and if/how they interact with the midgut cells of both species. A significant amount of Vip3Ab1 was clearly detected in the foregut and midgut regions of treated *S. frugiperda* larvae (Figure 6B). Some protein was detected in several goblet cell lumens and on some columnar cell remnants. However, due to the significant damage caused by this protein (Figure 5C), the precise location of Vip3Ab1 binding to the midgut cells could not be determined. Little to no staining of Vip3Ab1 was observed in the midgut of *S. eridania* (Figure 6D). Some minor staining of material in the foregut was noted on some larvae, presumably from Vip3Ab1 protein present in the food material. This lack of Vip3Ab1 staining in the midgut region is consistent with the lack of damage noted in the histopathological specimens (Figure 5G). 

Vip3Ab1-740 was detected in the foregut and midgut regions of intoxicated *S. frugiperda* larvae (Figure 6A) in a pattern and intensity similar to what was observed for Vip3Ab1 in *S. frugiperda* (Figure 6B). Here again, some Vip3Ab1-740 protein was localized in the lumens of some goblet cells, and light staining was noted on several columnar cells. As seen by histopathology, and similar to the Vip3Ab1-treated *S. frugiperda* larvae, the enterocytes of Vip3Ab1-740-treated *S. frugiperda* larvae were too badly damaged for subcellular localization determination. In *S. eridania*, some Vip3Ab1-740 was detected in the midgut lumen, in contrast to Vip3Ab1, which was not (Figure 6C,D). The signal intensity of Vip3Ab1-740 was much lower in the midgut of *S. eridania* (Figure 6C) compared to the signal in *S. frugiperda* midgut (Figure 3), and very little Vip3Ab1-740 protein was observed interacting with the midgut enterocytes in *S. eridania*. However, although there was less Vip3Ab1-740 protein in the *S. eridania* midgut, the dose used for these microscopy studies has been demonstrated to have a high mortality in *S. eridania* larvae in multiple bioassay studies, including in the range-finding studies done for these experiments (Supplementary Figures S2 and S3).

## 3. Discussion

Vip3A genes encode insecticidal proteins with a mode of action that is different than Cry proteins and are currently used to provide protection from a broad spectrum of lepidopteran crop pests [14]. In a companion study, we have shown that modification of the C-terminus of Vip3Ab1 can confer lethal activity towards *S. eridania*, a major threat to South American soybean crops that is not controlled by native Vip3Ab1 [20,21]. However, the factors that determine Vip3A specificity are poorly understood and, in all cases to date, no other Vip3A proteins have been reported to have differential activity within the genus of spodoptera [2,22,23,24,25,26]. Thus, the disparate activities of Vip3Ab1 and Vip3Ab1-740 towards *S. eridania* offer a unique opportunity to examine their insecticidal effects and biochemical behavior in two related insect species. This new information will be valuable towards understanding Vip3A specificity determinants and mechanism of action against spodopteran pests.

It has been reported that ~85 kDa full-length Vip3 proteins form tetramers that must be proteolytically processed to an active form prior to pore formation [3,4,5,14]. This processing results in ~65 kDa and ~20 kDa products, which remain associated to form an activated toxin tetramer presumed necessary for protein stability and insecticidal function [5]. Here, we investigated the processing of each protein by midgut proteases from *S. frugiperda* and *S. eridania* to determine if there was limitation in processing by *S. eridania*. We found no clear differences in Vip3A in vitro proteolytic processing from either insect species, indicating that Vip3Ab1 and Vip3Ab1-740 could be efficiently activated by midgut enzymes from either insect. However, overnight incubations suggested further processing of Vip3Ab1, as smaller molecular weight bands were apparent when visualized by SDS-PAGE. It should be noted that the midgut enzymes utilized in this study were normalized for total proteolytic activity and may not accurately reflect the specific level of proteolytic activity of the midgut lumen in vivo. We also cannot rule out the possibility that differences in pH in the midguts of the two species may have an effect. Kunthic et al. demonstrated a direct correlation between the pore-forming properties of Vip3Aa and the pH conditions [3]. A recent study by Abdelgaffar et al. found significant differences in pH and protease activity between midgut fluids from *H. virescens* and *H. zea* [27]. Regardless, these data indicate that both *S. frugiperda* and *S. eridania* midguts are equipped with enzymes capable of activating either Vip3A protein.

Because digestion analysis suggested that Vip3Ab1 was efficiently activated by midgut fluids of *S. eridania*, we hypothesized that Vip3Ab1 might be inactivated by enzymatic degradation in vivo. Banyuls et al. described a region of approximately 150 amino acids in the C-terminus critical for the proteolytic stability of Vip3 proteins, and that alterations within this region generally led to a loss of protein stability and activity [28]. We also previously demonstrated that large (~580 amino acids) C-terminal alterations can prevent the formation of tetramers, which are thought to be important for stability and insecticidal activity [5]. The chimeric protein utilized in this study, Vip3Ab1-740, contains a more modest C-terminal modification of 177 amino acids, and was therefore thought to be less likely to destabilize the protein. As expected, both Vip3Ab1-740 and Vip3Ab1 were visible as tetramers by both SDS-PAGE and size exclusion chromatography after processing by enzymes from *S. frugiperda* or *S. eridania*. However, overnight incubation with *S. frugiperda* or *S. eridania* midgut fluids revealed degradation of the Vip3Ab1 tetramers by analytical size exclusion chromatography. This level of degradation was not seen in Vip3Ab1-740 under the same conditions, indicating that Vip3Ab1-740 has increased stability relative to Vip3Ab1 in the presence *S. frugiperda* or *S. eridania* midgut fluids. This also suggests that midgut enzymes from either insect are capable of disrupting Vip3Ab1 tetramer formation.

We also briefly surveyed the enzymatic profile of gut enzymes isolated from *S. frugiperda* and *S. eridania*. There were no obvious differences in molecular weights of midgut proteases from *S. frugiperda* and *S. eridania*, as analyzed by zymogram. In addition, both *S. frugiperda* and *S. eridania* midgut fluids were shown to contain serine proteases, as demonstrated by the inhibition of Vip3Ab1 processing by chymostatin or benzamidine in vitro. This is not surprising, as several groups have shown the major proteinase activities in the lepidopteran midgut are serine proteinase-like enzymes [29,30,31]. Therefore, we conclude that the proteolytic environment is very similar between *S. frugiperda* and *S. eridania*, where both appear to be dominated by serine protease activity.

Because the processing of Vip3Ab1 and Vip3Ab1-740 was very similar, and the proteolytic activity found in midgut fluids from *S. frugiperda* and *S. eridania* were comparable, the differential toxicity of Vip3Ab1 could not be explained by these data. Therefore, we suspected that our in vitro analyses may not be entirely reflective of the in vivo environment. For example, proteolytic degradation may prevent Vip3Ab1 from reaching its target in the midgut epithelium of *S. eridania* in vivo. Shao et al. observed that midgut proteinase from *Helicoverpa armigera* contained robust proteolytic activity and hypothesized that this activity could inactivate Bt-derived delta endotoxins [31]. Therefore, we attempted to inhibit serine proteases in vivo with the broad-spectrum serine protease inhibitor, benzamidine. Preliminary bioassays confirmed benzamidine alone had no obvious toxicity, which is in agreement with other reports showing only modest effects of protease inhibitors on the growth and development of other lepidopteran pests [29,31]. We also confirmed that processing Vip3Ab1 in vitro with midgut enzymes had a relatively minor impact on insecticidal activity, as there was a 3–5 fold reduction in potency (LC_50_). Therefore, we anticipated that co-feeding larvae with processed Vip3Ab1 in the presence of benzamidine would effectively restore activity against *S. eridania*, as protein would be protected from proteolysis, thus allowing Vip3Ab1 to reach receptors on the midgut epithelium. However, we could not accurately test this hypothesis, as benzamidine completely protected *S. eridania* and *S. frugiperda* from insecticidal activity of either Vip3A protein, regardless of pre-processing. This result is in stark contrast to the synergistic effects of serine protease inhibitors observed with three different Cry1 family toxins on *Spodoptera exigua* [29]. In these studies, Ma et al. hypothesized that proteinase inhibitors protected the Cry toxins from excessive degradation in the midgut, leading to the observed increase in toxicity, and proposed that co-expression of Cry proteins with proteinase inhibitor genes would be an effective means to enhance potency against *S. exigua*. Similarly, Fortier et al. proposed that membrane-bound proteases inactivate Cry1Ab in the *Manduca sexta* midgut [32]. Several other studies have suggested similar means to improve Cry1 toxicity to lepidopteran pests [31,33,34,35]. It is possible that benzamidine directly modifies Vip3A proteins, making them unavailable to bind to midgut receptors; however, this is very unlikely, as benzamidine is a reversible competitor of serine proteases, does not form covalent bonds with serine residues, and has a specific interaction with the substrate binding pocket of trypsin-like proteases [36]. Therefore, our data suggests yet another distinguishing feature of Vip3A proteins is the potential reliance on serine proteases downstream of the initial proteolytic processing that generates ~65 kDa and ~20 kDa fragments found in active Vip3A tetramers for in vivo insecticidal activity. 

While our in vitro analyses indicated that the C-terminus of Vip3Ab1-740 conferred stability to protein tetramers, we were unable to utilize benzamidine as a means to prevent Vip3Ab1 degradation in vivo and test the importance of this improvement towards insecticidal activity on *S. eridania*. Therefore, we employed histopathology and immunohistochemistry to evaluate the cellular effects and the localization of each protein in vivo. We observed that after two days of exposure, susceptible larvae did not grow and/or molt and were considered moribund. The histopathological effects of both Vip3Ab1 and Vip3Ab1-740 were similar to those of Cry1Fa in susceptible insects, as the majority of cellular damage was observed in the midgut compartment, which is consistent with previous reports [8,37,38,39,40]. The midgut of *S. eridania* treated with Vip3Ab1 appeared similar to untreated controls. Specifically, the midgut cells were not damaged or in a state of active repair, suggesting that the lack of *S. eridania* susceptibility to Vip3Ab1 was not due to an accelerated or enhanced healing response. Immunolocalization confirmed the presence of both proteins in the foregut and midgut regions of *S. frugiperda*. Interestingly, while both Vip3Ab1 and Vip3Ab1-740 were clearly detected in the foregut of *S. eridania*, only Vip3Ab1-740 was detected in the midgut compartment in this species. Because we utilized a polyclonal antibody which has reactivity to Vip3Ab1 and Vip3Ab1-740 proteins, in both native and reducing/denaturing conditions, we hypothesize that the absence of Vip3Ab1 in the midgut of *S. eridania* is likely reflective of protein degradation rather than epitope loss or modification. 

The work described here provides a more thorough understanding of the mechanisms that govern the susceptibility of spodopteran pests to Vip3A. From these results, we conclude that it is an increased stability of Vip3Ab1-740 which confers insecticidal activity towards *S. eridania*, an important South American soybean pest. In reaching this conclusion, we have identified three key areas for future research: (1) Comparative analyses of the spodopteran midgut environments, (2) exploration of how the C-terminus impacts the stability of Vip3A proteins, and (3) further research into the mechanism by which benzamidine protects insects from Vip3A insecticidal activity.

## 4. Materials and Methods 

### 4.1. Gene and Protein Sequences 

The sequence for Vip3Ab1 corresponds to GenBank Accession AAR40284.1. DIG740 was identified from strain DBt11861 of our internal strain collection. This sequence was found to be identical to GenBank Accession KC156693.1, which corresponds to Vip3Ai1. 

### 4.2. Construct Design

To generate a chimeric Vip gene consisting of the first 1851 bp of Vip3Ab1 and the last 540 bp of DIG740, polymerase chain reactions (PCR) were performed to generate the two products, then a second round of PCR was performed to join the two products using overlapping PCR with the forward Vip3Ab1 primer and the reverse DIG740 primer. PCR was performed using Phire Hot Start II polymerase (Thermo Fisher Scientific, Waltham, MA, USA) in the following reaction: 27 μL H2O, 10 μL 5× Phire buffer, 1 μL dNTP mix, 5 μL Forward (10 μM), 5 μL Reverse (10 μM), 1 μL DIG307 (20 ng/ μL) and 1 μL Phire polymerase. Cycling was 98 °C/30 s followed by 30 cycles of 98 °C/5 s, 50.8 °C /5 s, 72 °C/2 min followed by a final extension at 72 °C/1 min then hold at 4 °C. For the assembly of the full length chimeric gene the following reaction was used: 26 μL H2O, 10 μL 5X Phire buffer, 1 μL dNTP mix, 5 μL Vip3Ab1 Forward (10 μM), 5 μL DIG740 Reverse (10 μM), 1 μL Part A reaction, 1 μL Part B reaction and 1 μL Phire polymerase. Cycling was as above, except using 2.5 min extension time. The 2397 bp product was gel purified and ligated into pCR-BluntII-TOPO (Thermo Fisher Scientific) and sequenced. A clone having the correct sequence was digested with BamHI and the fragment was gel purified. The fragment was ligated into pET24(+) (MilliporeSigma, Burlington, MA, USA) which was linearized with BamHI and rSAP treated using NEB T4 DNA ligase. Minipreps were performed and a clone having the gene in the proper orientation was selected for expression.

### 4.3. Protein Expression and Purification

Cry1Fa protein was expressed and purified from *Pseudomonas fluorescens* as previously described [41]. Vip3Ab1 and Vip3Ab1-740 were initially expressed in *E. coli* BL21 (DE3) cells. Briefly, seed and production cultures were grown in TB media (Fisher BioReagents™ Terrific Broth, Cat#BP9728-2) supplemented with 50 µg/mL of kanamycin (GoldBio, Cat#K-120-100). Production cultures were inoculated with a 1:200 dilution of an overnight seed culture (16–18 h) and incubated at 37 °C, 225 rpm, in a 2.5 cm throw shaker, until mid-log phase (OD600 0.6–0.9). Protein expression was then induced with the addition of 1 mM IPTG and incubated for 18–20 h at 18 °C, 200 rpm in a 2.5 cm throw shaker. Larger scale (>1 L) of both proteins used recombinant *P. fluorescens* strains as described previously [5]. Proteins purified from both systems have been shown to have equivalent potency, and the purification methods were the same. Vip3Ab1 was purified as previously described [5], and Vip3Ab1-740 was purified in a similar manner. Harvested cells containing Vip3Ab1-740 were sonicated in lysis buffer consisting of 50 mM Tris-HCl (pH 8.0), 1 M NaCl, 10% glycerol and 2 mM EDTA with 50 µL of protease inhibitor cocktail (Sigma-Aldrich, St. Louis, MO, USA) per 25 mL buffer. The extract was centrifuged at 20,000× *g* for 40 min. The soluble protein in the supernatant was precipitated with 50% ammonium sulfate and centrifuged at 20,000× *g* for 20 min. The pellet was resuspended in 50 mM Tris-HCl (pH 8.0) and purified by anion exchange chromatography using a HiTrap™ Q HP 5 mL column with an AKTA Purifier chromatography system (GE Healthcare, Chicago, IL, USA). The column was equilibrated in 50 mM Tris-HCl (pH 8.0), and proteins were eluted with a stepwise gradient to 1 M NaCl. Protein-containing fractions were combined and concentrated using Amicon® Ultra-15 Centrifugal Filter Devices with a 30 kDa MWCO (MilliporeSigma, Burlington, MA, USA). Proteins were desalted to 50 mM Tris-HCl (pH 8.0) using Zeba® Spin Desalting Columns, 7 MWCO (Thermo Scientific) or by dialysis in 50 mM Tris-HCl (pH 8.0) using Slide-A-Lyzer® Dialysis Cassettes, 20,000 MWCO (Thermo Scientific). Total protein concentrations were measured with the NanoDrop 2000C Spectrophotometer (Thermo Scientific), using the A280 method. DIG740 was expressed in *P. fluorescens* but was not purified due to lack of activity on insect bioassay.

### 4.4. SDS-PAGE Analysis

SDS-PAGE analysis was performed using NuPAGE® Novex® 4–12% Bis-Tris Protein Gels (Thermo Scientific). Proteins were diluted in 4× NuPAGE® LDS Sample Buffer (Thermo Scientific) containing 100 mM TCEP prior to loading onto the gel. Ten µL of Novex® Sharp Pre-stained Protein Standard (Thermo Scientific) was loaded onto one lane of each gel. Gels were run according to the manufacturer’s recommendations using NuPAGE® MES SDS Running Buffer (Thermo Scientific) and stained with SimplyBlue™ SafeStain (Thermo Scientific), then destained in water and imaged on a flatbed scanner.

### 4.5. Lepidopteran Midgut Fluid Protein Digestion and Analytical Size Exclusion Chromatography

Midguts were harvested from fifth instar larvae of *S. frugiperda* and *S. eridania* and stored at approximately −80 °C. Midgut fluids were extracted by vortexing in 8.5% sucrose solution with 150 mM NaCl followed by centrifugation at 10,000× *g* for 10 min at 4 °C. Prepared midgut fluids were aliquoted and stored at approximately −80 °C. Midgut fluids were normalized by total proteolytic activity using BODIPY-casein degradation assay as previously described [5]. Proteins were added to reactions at 150 µg/mL final concentration. All digestions were performed at both pH 8.0 and pH 10.0. Control reactions were prepared containing no insect gut fluid. Reactions were incubated with shaking at 30 °C for various time intervals, and protease inhibitor cocktail (Sigma-Aldrich) was added to terminate the reactions prior to SDS-PAGE analysis. Analytical size exclusion chromatography was performed as previously described [5], and injection volumes were six µL at a protein concentration of 150 µg/mL. Estimated sizes of protein species were calculated based on protein molecular weight standards from Sigma-Aldrich (#69385).

### 4.6. Protease Inhibitors Used in Midgut Fluid Digestions and Insect Bioassay

Selected protease inhibitors were added to digestion reactions in order to define the types of proteases present in midgut fluids. Bestatin and chymostatin (Roche Diagnostics, Indianapolis, IN, USA) were resuspended in methanol and DMSO respectively according to the manufacturer’s recommendations and added to reactions at final concentrations of 130 µM and 100 µM respectively. Benzamidine hydrochloride hydrate (Sigma-Aldrich) was resuspended in 50 mM Tris-HCl (pH 8.0) and added to reactions at a final concentration of 5 mM. For insect bioassays, benzamidine was added directly to the prepared proteins in 50 mM Tris-HCl (pH 8.0) at final concentrations of 9 mM, 3 mM, 1 mM and 0.3 mM relative to the diet volume of 1.5 mL per well.

#### 4.6.1. Zymogram Analysis

Midgut fluids were analyzed for protease banding patterns using a Novex 12% Zymogram (Casein) Gel (Thermo Scientific). *S. frugiperda* and *S. eridania* midgut fluids were combined with water and Tris-Glycine sodium dodecyl-sulfate (SDS) Sample Buffer (Thermo Scientific) and loaded onto the gel in 10-fold serial dilutions. The gel was run and developed according to the manufacturer’s recommendations followed by staining and imaging as above.

#### 4.6.2. Insect Diet Overlay Bioassays 

Bioassays were conducted essentially as previously described [5]. All statistical analyses were conducted using JMP 12.2 software (SAS Software). Probit analyses of the pooled mortality and moribund data were used to estimate the 50% lethal concentration (LC_50_). Binomial distribution analysis was used to generate 95% confidence intervals for comparison of benzamidine co-feeding effects.

### 4.7. Histopathology

Initial range finding studies were performed to determine a time point and a dose at which the effects of both Vip3A proteins could be visualized in treated insects, but prior to death. At 600 ng/cm^2^, a majority of larvae were dead after three days of feeding (Supplementary Figures S2 and S3); therefore, living larvae were collected after two days exposure to this level of toxin. One hundred and twenty five insects were treated with insecticidal proteins for two days to allow multiple living larvae to be collected for histopathology. 

Neonate *S. frugiperda* and *S. eridania* larvae (<24 h old) were exposed to insecticidal proteins, or buffer overlaid on artificial diet in 48-well microplates. Vip3Ab1 or Vip3Ab1-740 was applied at 600 ng/cm^2^; Cry1F was applied at 1200 ng/cm^2^. Larvae were allowed to feed on this diet for 48 h. Larvae were fixed overnight at 4 °C in 4% formaldehyde; 0.01% Silwet L-77; in PBS, pH 7.4. Anterior and posterior regions of each animal were removed, and dissected larvae were then fixed for an additional 24 h at 4 °C. Larvae were dehydrated with a graded ethanol series, infiltrated in a graded series of LR white resin, moved to flat-bottomed polyethylene capsules (TAAB, EMS), and polymerized at 50 °C for 3 h. Larvae with minimal axial curvature were selected and re-polymerized into flat-bottomed plastic capsules in a lateral orientation to achieve the optimal sectioning plane. A series of sections (500 nm thick) was made with a diamond knife on a Leica UC7 ultramicrotome through each of the larvae (depth series). Sections were stained with toluidine blue O at 60 °C for 1 min and mounted with Polymount-xylene (Polysciences). Slides were observed and imaged with a Leica DM5000 upright microscope. The sections showing the most ideal median plane of the anterior-most region of the midgut were photographed. Figure panels were made using the GIMP.

### 4.8. Immunolocalization

For immunolocalization studies, larvae were dissected and fixed as above, dehydrated, and embedded in graded 3:1 butyl/methyl methacrylate (Polysciences) and polymerized at 4 °C with UV light. One micron thick sections were cut with a Diatome histo knife, dried onto Fisher Plus slides, and de-plasticized with a 10 min incubation in acetone. A rabbit polyclonal antibody was raised to purified Vip3Ab1 protein. Due to the high degree of similarity between Vip3Ab1 and Vip3Ab1-740, the same antibody was used to recognize both proteins. Experiments were conducted to determine the appropriate antibody diluent, epitope retrieval conditions, and antibody concentration. Ideal conditions were found that gave strong signal on treated larvae sections, and no appreciable reaction on untreated sections. An HRP-conjugated secondary antibody was used for detection, followed by Discovery Silver chromogen (Roche Diagnostics). Sections were imaged with a combination of autofluorescence (tissue) and confocal reflection (silver) on a Leica SP5 LSCM. 

## Figures and Tables

**Table 1:** Comparison of in vitro midgut enzyme processed and full length Vip3Ab1 and Vip3Ab1-740 against *S. frugiperda*. Processed Vip3 samples were generated by 24 h digestion with midgut fluids at pH 8.0 to allow for complete processing. LC_50_ concentration was calculated using a Probit analysis of the sum of dead and moribund insects relative to the total number of treated insects.

Protein	Vip3Ab1				Vip3Ab1-740			
	**LC_50_ (ng/cm^2^)**	**Lower CI (95%)**	**Upper CI (95%)**	**N**	**LC_50_ (ng/cm^2^)**	**Lower CI (95%)**	**Upper CI (95%)**	**N**
**Full length**	177.9	138.6	230.7	362	80.8	69.0	94.6	1056
**Processed**	590.2	468.4	739.0	224	435.0	368.6	552.4	224

N = total number of insects tested per protein; CI = confidence interval at 95%.

## Supplementary Materials

The following are available online at https://www.mdpi.com/2072-6651/11/5/276/s1, Figure S1: The proteases present in *S. frugiperda* and *S. eridania* midgut fluids were analyzed on a casein zymogram, Figure S2: Range finding bioassay for *S. frugiperda*, Figure S3: Range finding bioassay for *S. eridania*.

## References

[1] Chakroun M., Banyuls N., Bel Y., Escriche B., Ferre J. (2016). Bacterial Vegetative Insecticidal Proteins (Vip) from Entomopathogenic Bacteria. Microbiol. Mol. Biol. Rev..

[2] Estruch J.J., Warren G.W., Mullins M.A., Nye G.J., Craig J.A., Koziel M.G. (1996). Vip3A, a novel Bacillus thuringiensis vegetative insecticidal protein with a wide spectrum of activities against lepidopteran insects. Proc. Natl. Acad. Sci. USA.

[3] Kunthic T., Watanabe H., Kawano R., Tanaka Y., Promdonkoy B., Yao M., Boonserm P. (2017). pH regulates pore formation of a protease activated Vip3Aa from Bacillus thuringiensis. Biochim. Biophys. Acta.

[4] Palma L., Scott D.J., Harris G., Din S.U., Williams T.L., Roberts O.J., Young M.T., Caballero P., Berry C. (2017). The Vip3Ag4 Insecticidal Protoxin from Bacillus thuringiensis Adopts A Tetrameric Configuration That Is Maintained on Proteolysis. Toxins (Basel).

[5] Zack M.D., Sopko M.S., Frey M.L., Wang X., Tan S.Y., Arruda J.M., Letherer T.T., Narva K.E. (2017). Functional characterization of Vip3Ab1 and Vip3Bc1: Two novel insecticidal proteins with differential activity against lepidopteran pests. Sci. Rep..

[6] Anilkumar K.J., Rodrigo-Simon A., Ferre J., Pusztai-Carey M., Sivasupramaniam S., Moar W.J. (2008). Production and characterization of Bacillus thuringiensis Cry1Ac-resistant cotton bollworm Helicoverpa zea (Boddie). Appl. Environ. Microbiol..

[7] Ben Hamadou-Charfi D., Boukedi H., Abdelkefi-Mesrati L., Tounsi S., Jaoua S. (2013). Agrotis segetum midgut putative receptor of Bacillus thuringiensis vegetative insecticidal protein Vip3Aa16 differs from that of Cry1Ac toxin. J. Invertebr. Pathol..

[8] Chakroun M., Ferre J. (2014). In vivo and in vitro binding of Vip3Aa to Spodoptera frugiperda midgut and characterization of binding sites by (125)I radiolabeling. Appl. Environ. Microbiol..

[9] Gouffon C., Van Vliet A., Van Rie J., Jansens S., Jurat-Fuentes J.L. (2011). Binding Sites for Bacillus thuringiensis Cry2Ae Toxin on Heliothine Brush Border Membrane Vesicles Are Not Shared with Cry1A, Cry1F, or Vip3A Toxin. Appl. Environ. Microb..

[10] Lee M.K., Miles P., Chen J.S. (2006). Brush border membrane binding properties of Bacillus thuringiensis Vip3A toxin to Heliothis virescens and Helicoverpa zea midguts. Biochem. Biophys. Res. Commun..

[11] Welch K.L., Unnithan G.C., Degain B.A., Wei J., Zhang J., Li X., Tabashnik B.E., Carriere Y. (2015). Cross-resistance to toxins used in pyramided Bt crops and resistance to Bt sprays in Helicoverpa zea. J. Invertebr. Pathol..

[12] Horikoshi R.J., Bernardi D., Bernardi O., Malaquias J.B., Okuma D.M., Miraldo L.L., Amaral F.S., Omoto C. (2016). Effective dominance of resistance of Spodoptera frugiperda to Bt maize and cotton varieties: implications for resistance management. Sci. Rep..

[13] Huang F., Qureshi J.A., Meagher R.L., Reisig D.D., Head G.P., Andow D.A., Ni X., Kerns D., Buntin G.D., Niu Y. (2014). Cry1F resistance in fall armyworm Spodoptera frugiperda: Single gene versus pyramided Bt maize. PLoS ONE.

[14] Lee M.K., Walters F.S., Hart H., Palekar N., Chen J.S. (2003). The mode of action of the Bacillus thuringiensis vegetative insecticidal protein Vip3A differs from that of Cry1Ab delta-endotoxin. Appl. Environ. Microbiol..

[15] Martinelli S., Barata R.M., Zucchi M.I., Silva-Filho Mde C., Omoto C. (2006). Molecular variability of Spodoptera frugiperda (Lepidoptera: Noctuidae) populations associated to maize and cotton crops in Brazil. J. Econ. Entomol..

[16] Omoto C., Bernardi O., Salmeron E., Sorgatto R.J., Dourado P.M., Crivellari A., Carvalho R.A., Willse A., Martinelli S., Head G.P. (2016). Field-evolved resistance to Cry1Ab maize by Spodoptera frugiperda in Brazil. Pest Manag. Sci..

[17] Storer N.P., Babcock J.M., Schlenz M., Meade T., Thompson G.D., Bing J.W., Huckaba R.M. (2010). Discovery and characterization of field resistance to Bt maize: Spodoptera frugiperda (Lepidoptera: Noctuidae) in Puerto Rico. J. Econ. Entomol..

[18] Chakroun M., Banyuls N., Walsh T., Downes S., James B., Ferre J. (2016). Characterization of the resistance to Vip3Aa in Helicoverpa armigera from Australia and the role of midgut processing and receptor binding. Sci. Rep..

[19] Wu J., Zhao F., Bai J., Deng G., Qin S., Bao Q. (2007). Evidence for positive Darwinian selection of Vip gene in Bacillus thuringiensis. J. Genet. Genomics..

[20] Sopko M.S., Narva K.E., Bowling A.J., Pence H.E., Hasler J.M., Letherer T.J., Larsen C.L., Zack M.D. (2019). Modification of Vip3Ab1 C-terminus confers broadened plant protection from lepidopteran pests. Toxins.

[21] Murua M.G., Vera M.A., Herrero M.I., Fogliata S.V., Michel A. (2018). Defoliation of Soybean Expressing Cry1Ac by Lepidopteran Pests. Insects.

[22] Bergamasco V.B., Mendes D.R., Fernandes O.A., Desiderio J.A., Lemos M.V. (2013). Bacillus thuringiensis Cry1Ia10 and Vip3Aa protein interactions and their toxicity in Spodoptera spp. (Lepidoptera). J. Invertebr. Pathol..

[23] Chakroun M., Bel Y., Caccia S., Abdelkefi-Mesrati L., Escriche B., Ferre J. (2012). Susceptibility of Spodoptera frugiperda and S. exigua to Bacillus thuringiensis Vip3Aa insecticidal protein. J. Invertebr. Pathol..

[24] Chen J., Yu J., Tang L., Tang M., Shi Y., Pang Y. (2003). Comparison of the expression of Bacillus thuringiensis full-length and N-terminally truncated vip3A gene in Escherichia coli. J. Appl. Microbiol..

[25] Palma L., de Escuder I.R., Maeztu M., Caballero P., Munoz D. (2013). Screening of vip genes from a Spanish Bacillus thuringiensis collection and characterization of two Vip3 proteins highly toxic to five lepidopteran crop pests. Biol. Control..

[26] Ruiz de Escudero I., Banyuls N., Bel Y., Maeztu M., Escriche B., Munoz D., Caballero P., Ferre J. (2014). A screening of five Bacillus thuringiensis Vip3A proteins for their activity against lepidopteran pests. J. Invertebr. Pathol..

[27] Abdelgaffar H.M., Oppert C., Sun X., Monserrate J., Jurat-Fuentes J.L. (2019). Differential heliothine susceptibility to Cry1Ac associated with gut proteolytic activity. Pestic. Biochem. Physiol..

[28] Banyuls N., Hernandez-Rodriguez C.S., Van Rie J., Ferre J. (2018). Critical amino acids for the insecticidal activity of Vip3Af from Bacillus thuringiensis: Inference on structural aspects. Sci. Rep..

[29] Ma Y., Zhang Y., Chen R.R., Ren X.L., Wan P.J., Mu L.L., Li G.Q. (2013). Combined effects of three crystalline toxins from Bacillus thuringiensis with seven proteinase inhibitors on beet armyworm, Spodoptera exigua Hubner (Lepidoptera: Noctuidae). Pestic. Biochem. Phys..

[30] Purcell J.P., Greenplate J.T., Sammons R.D. (1992). Examination of Midgut Luminal Proteinase Activities in 6 Economically Important Insects. Insect Biochem. Molec..

[31] Shao Z.Z., Cui Y.L., Liu X.L., Yi H.Q., Ji J.H., Yu Z.N. (1998). Processing of delta-endotoxin of Bacillus thuringiensis subsp. kurstaki HD-1 in Heliothis armigera midgut juice and the effects of protease inhibitors. J. Invertebr. Pathol..

[32] Fortier M., Vachon V., Frutos R., Schwartz J.L., Laprade R. (2007). Effect of insect larval midgut proteases on the activity of Bacillus thuringiensis Cry toxins. Appl. Environ. Microbiol..

[33] Pardo-Lopez L., Munoz-Garay C., Porta H., Rodriguez-Almazan C., Soberon M., Bravo A. (2009). Strategies to improve the insecticidal activity of Cry toxins from Bacillus thuringiensis. Peptides.

[34] Zhu Y.C., Guo Z.B., Abel C. (2012). Cloning eleven midgut trypsin cDNAs and evaluating the interaction of proteinase inhibitors with Cry1Ac against the tobacco budworm, Heliothis virescens (F.) (Lepidoptera: Noctuidae). J. Invertebr. Pathol..

[35] Zhu Y.C., West S., Liu F.X., He Y.P. (2012). Interaction of proteinase inhibitors with Cry1Ac toxicity and the presence of 15 chymotrypsin cDNAs in the midgut of the tobacco budworm, Heliothis virescens (F.) (Lepidoptera: Noctuidae). Pest Manag. Sci..

[36] Krieger M., Kay L.M., Stroud R.M. (1974). Structure and specific binding of trypsin: comparison of inhibited derivatives and a model for substrate binding. J. Mol. Biol..

[37] Abdelkefi-Mesrati L., Boukedi H., Chakroun M., Kamoun F., Azzouz H., Tounsi S., Rouis S., Jaoua S. (2011). Investigation of the steps involved in the difference of susceptibility of Ephestia kuehniella and Spodoptera littoralis to the Bacillus thuringiensis Vip3Aa16 toxin. J. Invertebr. Pathol..

[38] Abdelkefi-Mesrati L., Boukedi H., Dammak-Karray M., Sellami-Boudawara T., Jaoua S., Tounsi S. (2011). Study of the Bacillus thuringiensis Vip3Aa16 histopathological effects and determination of its putative binding proteins in the midgut of Spodoptera littoralis. J. Invertebr. Pathol..

[39] Sellami S., Cherif M., Jamoussi K. (2016). Effect of adding amino acids residues in N- and C-terminus of Vip3Aa16 (L121I) toxin. J. Basic. Microbiol..

[40] Yu C.G., Mullins M.A., Warren G.W., Koziel M.G., Estruch J.J. (1997). The Bacillus thuringiensis vegetative insecticidal protein Vip3A lyses midgut epithelium cells of susceptible insects. Appl. Environ. Microbiol..

[41] Bel Y., Sheets J.J., Tan S.Y., Narva K.E., Escriche B. (2017). Toxicity and Binding Studies of Bacillus thuringiensis Cry1Ac, Cry1F, Cry1C, and Cry2A Proteins in the Soybean Pests Anticarsia gemmatalis and Chrysodeixis (Pseudoplusia) includens. Appl. Environ. Microbiol..

