# Supplementary material for: Insecticidal Activity of a Vip3Ab1 Chimera Is Conferred by Improved Protein Stability in the Midgut of Spodoptera eridania
Source: Toxins (Basel). 2019 May 16;11(5):276. doi: 10.3390/toxins11050276 (PMC6563307; doi:10.3390/toxins11050276)
Supplement: Supplementary file 1 [file toxins-11-00276-s001.pdf]

Supplementary Materials: Insecticidal activity of a Vip3Ab1 chimera is conferred by improved protein stability in the midgut of Spodoptera eridania

Andrew J. Bowling, Megan S. Sopko, Sek Yee Tan, Cory M. Larsen, Heather E. Pence and Marc D. Zack

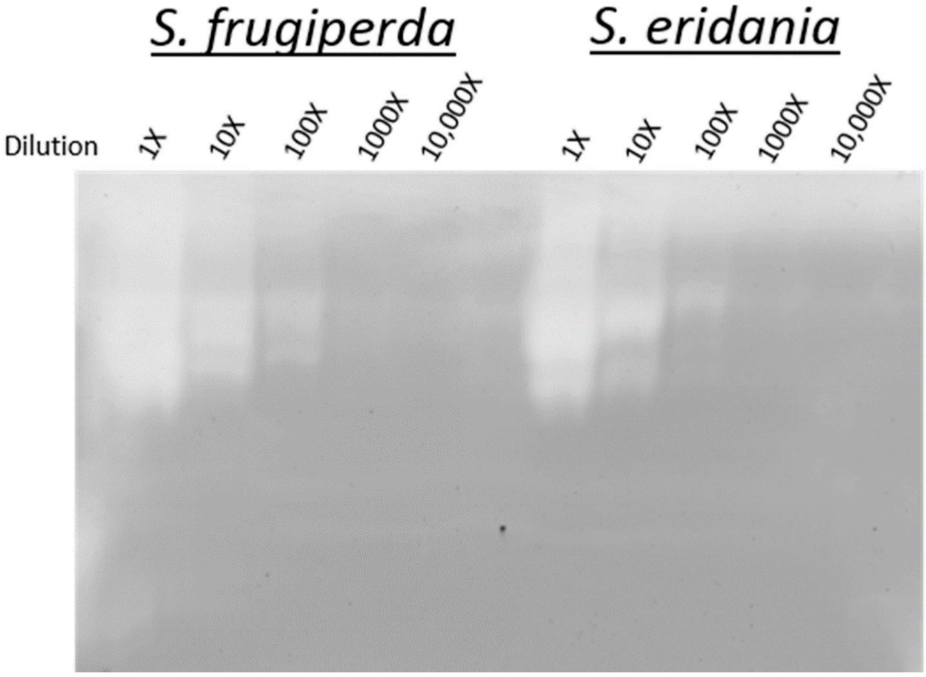

Figure S1. The proteases present in *S. frugiperda* and *S. eridania* midgut fluids were analyzed on a casein zymogram. Ten-fold serial dilutions were loaded onto the gel from 1X to 10,000X. Both species show similar patterns of negatively stained bands, which represent proteases.

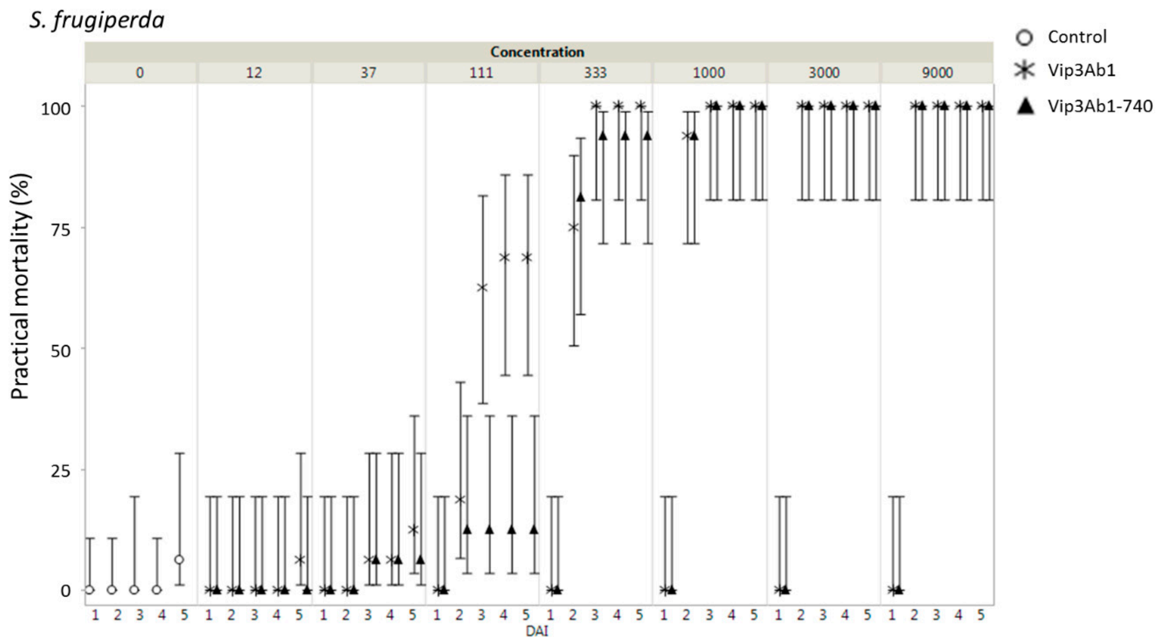

Figure S2. Range finding bioassay for *S. frugiperda*. Bioassays were performed using 16 insects at 7 different concentration of Vip3Ab1 or Vip3Ab1-740. The number of dead and moribund insects was recorded daily for 5 days after infestation (DAI). Percent practical mortality and associated 95% confidence intervals are shown.

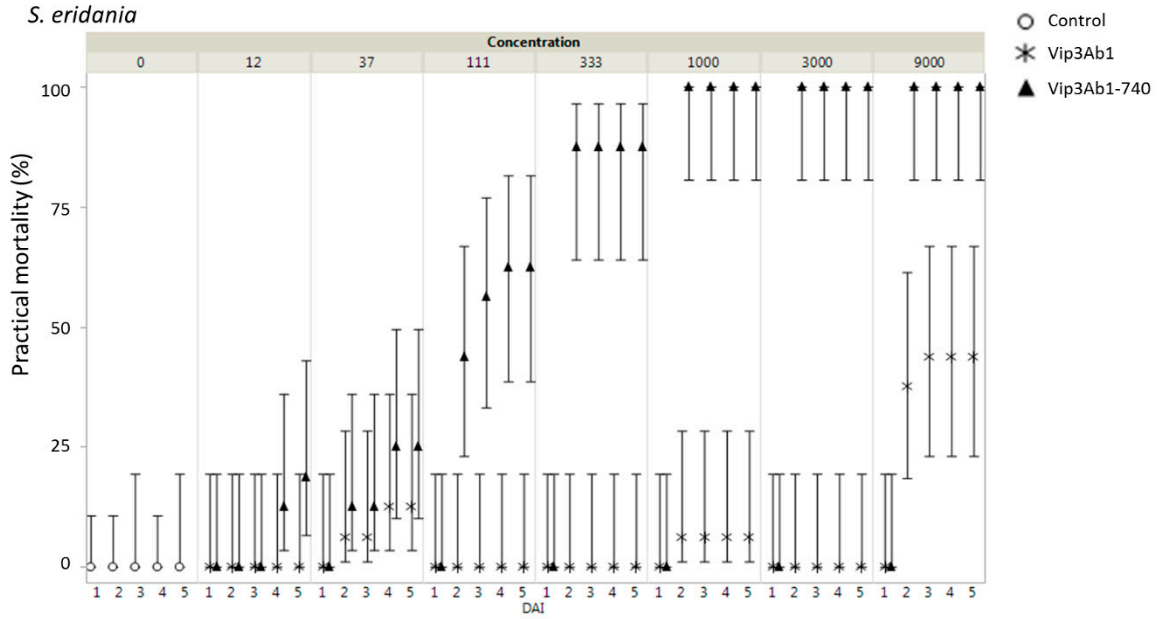

Figure S3. Range finding bioassay for *S. eridania*. Bioassays were performed using 16 insects at 7 different concentration of Vip3Ab1 or Vip3Ab1-740. The number of dead and moribund insects was recorded daily for 5 days after infestation (DAI). Percent practical mortality and associated 95% confidence intervals are shown.
